# Grasha-Richmann college students’ learning styles of classroom participation: role of gender and major

**Published:** 2014-07

**Authors:** ALI REZA BANESHI, MAHNAZ DEHGHAN TEZERJANI, HASAN MOKHTARPOUR

**Affiliations:** 1 Department of psychology and education, Tehran University, Tehran, Iran;; 2 Kharazmi University, Tehran, Iran;; 3 Allameh Tabatabaei University, Tehran, Iran

**Keywords:** Learning, participation, students

## Abstract

**Introduction:** This study aimed to investigate the male and female students’ learning styles of classroom participation and these styles’ differences between Humanities and Science majors.

**Method:** 1039 individuals were selected through the proportional stratified random sampling method among undergraduate and graduate students in Humanities (n=421) and Science (n=618) faculties of Tehran University. In the Humanities group, there were 285 females and 136 males, and in the Science group, there were 208 females and 410 males. The participants answered the Grasha-Riechmann student learning styles scale.

**Results:** The findings indicated that the females obtained significantly higher means in collaborative, participative, and dependent styles than males, but in avoidant, and independent styles, the means for males were higher than those for females. Also, the science group’s means in collaborative, participative, dependent, and competitive styles were significantly higher than those for the humanities group.

**Conclusion:** According to the findings, it seems that due to psychological characteristics, female students tend to collaborate with other students of the same sex and participate in their activities. In this way, they also are more dependent on their teacher and classroom, because otherwise they will face some problems such as anxiety. In addition, it seems that science students in comparison to humanities students are more participative and collaborative because they need more collaboration in their projects and course work.

## Introduction

In learning and teaching areas, the attempt has always been made to identify effective factors in learning such as individual differences to take necessary steps to improve it. According to Ackerman, Sternberg, and Glaser (1989), the review of literature suggests that two specific categories of predictor variables have been studied to explain learning and individual differences. Sarter and Jones state that the first category includes cognitive measures and ability tests (1). The second category of measures which is used to predict differences among students is non-cognitive measures. These measures include thinking styles (2, 3) and learning styles (1). In new psychological approaches, non-cognitive factors as well as learning processes are more emphasized than learning product and individuals’ abilities. In order to better understand the learning processes and of the factors which influence learning, psychology researchers have shifted their orientation from individuals’ fixed abilities and characteristics (intelligence) to non-cognitive factors (4).

In many cases, the individual’s preferred learning style which is known as learning style can be the cause of differences among learners. Learning styles are considered by some scholars (5-7) as an influencing factor on the learners’ educational performance. It is important to identify learners’ learning styles for leading teaching and learning activities, because it can help teachers to teach and treat their students with respect to the students’ certain characteristics and it can make learning more effective. Anderson and Adams (1992) consider identifying differences among learners as one of the most significant challenges that educators encounter. They also state that many teachers may not really understand the students’ differences in understanding knowledge and information. According to them, assuming the same cognitive skills for all students leads to the ignorance of the differences in learning styles (8).

There have been different theories of learning styles, most of which are cognitive styles (impulsive – reflective, Kolb's learning styles, etc.). Keefe (1979) classify these styles into five categories: physiological, attentional, receptive, expectancy and incentive, concept formation and retention. Sternberg and Grigorenko (1997) classify them into four categories: cognitive-oriented, personality-oriented, activity-oriented, and mental self-government styles (9).

Initially, it was assumed that in some of cognitive styles (reflective vs. impulsive) individuals act differently to solve issues due to their styles, but different studies have shown that acting reflectively or impulsively is influenced by the prior knowledge not the style itself (6) and thus, it doesn’t fit the definition of style as the preference. Accordingly, Grasha and Riechmann provide a model of learning styles which considers students’ interaction and participation instead of cognition and personality and thus, this model isn’t placed in the mentioned classifications. They believe that this model helps teachers and professors to recognize teaching which is appropriate for specific learning styles.

Grasha and Riechmann (1996) consider learning styles as social interactions and they define them as different roles that students have in interaction with classmates, teachers and course content (10). They suggest that learning styles can be identified through social and emotional dimensions such as attitudes toward learning, teachers, classmates and classroom. They present a model based on students' responses to the real classroom activities, not on the overall assessment of the personality (because the personality is constant, while styles are individuals’ preferences) or cognitive characteristics.

Grasha and Riechmann classify learning styles into six categories, each of which has its own characteristics. Individuals with avoidant style don’t like to be present in the classroom and don’t participate in the activities other students and the teacher do in the classroom. In general, they don’t enjoy the classroom climate and whatever is happening in the classroom. Individuals with participative style follow the class and enjoy going to and participating in the class so that they are eager to volunteer for activities and prefer to have discussion and lecture in the classroom. Individuals with collaborative style feel that learning is possible through sharing the ideas and opinions with stronger students and as a result, they interact with the teacher and would like to work with others and also prefer to discuss in small groups in the classroom. Individuals with dependent style have little curiosity for new learning and learn only what they are told to. They also consider teacher and classmates as resources for support and help and they are dependent on authorities to determine the area of activities. Individuals with independent style like to think by themselves and they are sure that they have the ability to learn. They prefer to learn the content which they think is important. Individuals with competitive style learn the content with the aim of having better performance than the other students in the classroom. These students believe that they have to compete with other students in the classroom to get reward.

Studies done in relation to Grasha-Riechmann learning styles (11-13)have shown that males and females have different learning styles, due to the gender characteristics. Also, the difference between learning styles can be due to the content of the study. For example Mahamod, et al. (2010) show that art students have a tendency toward collaborative and participative learning, while science students prefer independent learning (13) and Clark and Latshaw (2012) also state that students of different majors have different learning styles (14). Actually, it seems logical to expect different learning styles in different fields. Since the cultures and personality characteristics of each society are different, and thus students of that society use unique learning styles, the purpose of this study is to examine the styles preferred by males and females, and also, to examine the difference between science and the humanities majors due to their different contents and need for various learning styles.

## Methods

The methodology used in this study was non-experimental and ex-post-facto. The population of this study consisted of undergraduate and graduate students (N=15893) of Tehran University in 2011-12. In order to get the study sample, the stratified random sampling method was used; therefore, first faculties were classified to science and humanities (psychology, social sciences, management …) and then, 33 individuals were selected from each group. Due to variance in each group and 0.05 error rate, Z-value (=1.96 for 95% confidence level), σ^2^wx=total variance (3.39), x̄ =total mean (3.6), the sample size was calculated using the following formula.

$$n\geq \frac{\text{N}{\text{Z}}^{2}\frac{\sigma^{2}\text{wx}}{{\overset{-}{\text{x}}}^{2}}}{\text{N}{Ε}^{2}+{\text{Z}}^{2}\frac{\sigma^{2}\text{wx}}{{\overset{-}{\text{x}}}^{2}}}=\mathrm{760}$$

$$\sum =\text{error}\left(\text{0.05}\right)$$

$$\text{Z}=\text{Z}\;\text{ value }\left(=\text{1.96 for 95\% confidence level}\right)$$

N=population size(15893)

$$\sigma^{2}\text{w}=\text{total variance (3.39)}$$

$$\overset{-}{\text{x}}=\text{total mean (3.6)}$$

$$\frac{\sigma^{2}\text{wx}}{{\text{x}}^{2}}=\text{relative variance}$$

The minimum sample size was 760 (416 from science faculty and 344 from humanities faculty). Because of the big sample needed for factor analysis of Grasha-Riechmann Learning Styles Scale, 1200 were selected. Then, the questionnaires which weren't answered appropriately were omitted. So, 1039 (421 humanities and 618 science) students remained for the study analysis. In the humanities group, there were 285 females and 136 males, and in the science group, there were 208 female and 410 male students.

Grasha and Riechmann (1996) developed a scale of 60 items with six subscales (independent (10 items), dependent (10 items), avoidant (10 items), participative (10 items), competitive (10 items), and collaborative (10 items)) to identify the learning styles of the students. The answers were on a 5-degree Likert scale from strongly agree to strongly disagree. Psychometric characteristics of this scale were examined and confirmed in Iran. The internal consistency of the scale was calculated and Cronbach alpha for independent, dependent, avoidant, participative, competitive, and collaborative styles were respectively 0.58, 0.71, 0.75, 0.80, 0.77, 0.74. Factor analysis of this scale indicated that the scale had construct validity (13).

Data collection was conducted in the second half of 2011-12. After talking with professors and getting their permission, we explained the overall aim of the study to the participants (We want to know what ideas you have about some of the educational issues). Then they were told if anyone did not want to answer the questionnaire, they could express their opinion. They were also told that before answering the questions of the questionnaire, they should read the instructions carefully, and then mark the statements on their answer sheets with respect to their actual ideas. They were asked not to ask a question of the researcher and the others and to complete the questionnaire by themselves. After 20 minutes, which was determined for answering the questions, the questionnaires were collected and the participants were appreciated for their cooperation in the study.  

Descriptive statistics (mean, and standard deviation), and Hotelling’s T^2^ were used to analyze the data (p<0.05). 

## Results

Univariate analysis of variance (ANOVA) was used to examine the difference between learning styles. Descriptive statistics of male and female groups, and humanities and science groups are reported in Table 1.

**Table 1:** Descriptive statistics

**Row**	**Styles **	**Humanities** **Mean±SD**	**Science** **Mean±SD**	**Male** **Mean±SD**	**Female** **Mean±SD**
**1**	**Avoidant**	24.87±5.56	24.55** ± **5.80	25.39** ± **5.54	24.02** ± **5.71
**2**	**Collaborative**	34.95±5.07	36.63** ± **4.92	34.96** ± **5.19	36.38** ± **4.84
**3**	**Participative**	25.68±5.19	27.30** ± **4.73	25.57** ± **5.11	27.18** ± **4.90
**4**	**Dependent**	32.37±4.07	32.91** ± **3.97	32.03** ± **4.28	33.21** ± **3.66
**5**	**Competitive**	25.78±5.25	27.28** ± **4.96	26.14** ± **5.21	26.66** ± **5.14
**6**	**Independent**	28.52±4.17	28.28** ± **4.07	28.93** ± **4.18	27.86** ± **4.01

Hotelling’s T^2^ test assumptions (M-box = 46.92, F = 2.14; sphericity X^2^ = 1488.6, df = 20) were examined and confirmed. Wilks’ Lambda multivariate test with a value of 0.94 indicated a significant difference between males and females (p<0.01). As Table 2 shows, there was a significant difference between males and females in avoidant, participative, collaborative, dependent and independent styles, while there wasn’t a significant difference in competitive style. According to the means reported in Table 1, males had a higher mean in avoidant and independent styles, while females’ mean was higher in participative, collaborative, and dependent styles.

**Table 2:** Tests of between subject effects for gender

**Effect source**	**Dependent variable**	**SS**	**MS**	**F_(1, 1037)_**	**p**	**Effect size**
**Gender**	**Avoidant**	487.54	487.54	15.43	<0.010	0.010
**Error**		32765.23	31.60			
**Gender**	**Collaborative**	517.46	517.46	20.48	<0.010	0.020
**Error**		26195.70	25.26			
**Gender**	**Participative**	672.96	672.96	26.82	<0.010	0.030
**Error**		26023.65	25.10			
**Gender**	**Dependent**	361.75	361.75	22.66	<0.110	0.020
**Error**		16556.81	15.97			
**Gender**	**Competitive**	69.74	69.74	2.60	<0.110	0.001
**Error**		27817.12	26.82			
**Gender**	**Independent**	293.31	293.31	17.48	<0.110	0.020
**Error**		17404.14	16.78			

N=1039

Also to examine the differences in learning styles of participation between humanities and science groups, Hotelling’s T^2^ test assumptions (M-box = 46.92, F = 2.14; sphericity X^2^ = 1488.6, df =20) were confirmed. Wilks’ Lambda multivariate statistic with a value of 0.95 was also significant (p<0.01). As shown in Table 3, in collaborative, participative, dependent, and competitive styles, there was a significant difference between humanities and science groups, but there wasn’t any significant difference in avoidant and independent styles. According to the means reported in Table 1, science group had higher mean in collaborative, participative, dependent, and competitive styles than humanities group.

**Table 3:** Tests of between subject effects for major

**Effect source**	**Dependent variable**	**SS**	**MS**	**F_(1, 1037)_**	**p**	**Effect size**
**Major**	**Avoidant**	26.83	26.83	0.84	<0.360	0.001
**Error**		33225.94	32.04			
**Major**	**Collaborative**	706.78	706.78	28.18	<0.001	0.030
**Error**		26006.37	25.08			
**Major**	**Participative**	658.50	658.50	26.23	<0.001	0.020
**Error**		26038.11	25.11			
**Major**	**Dependent**	73.41	73.41	4.52	<0.030	0.000
**Error**		16845.14	16.24			
**Major**	**Competitive**	563.49	563.49	21.39	<0.001	0.020
**Error**		27323.37	26.35			
**Major**	**Independent**	14.11	14.11	0.83	<0.360	0.000
**Error**		17683.35	17.05			

N=1039

## Discussion

The purpose of this study was to investigate the differences in Grasha-Riechmann learning styles among students of humanities and science, as well as to examine the learning styles of males and females. The results indicated that males had significantly a higher mean in independent, and avoidant styles, while females’ mean was higher in cooperative, participative, and dependent styles. In respect to the field of education (major), the science group’s means in cooperative, participative, dependent and competitive styles were higher  than those for the humanities group.

According to the results, females have more desire to do thing which need more communication with others and generally they are more satisfied with communication and collaboration than males. On the other hand, males have a desire to make decisions and to do things more individually and they have a less tendency toward collaboration and dependence than females and therefore, have independent styles. Also, avoidant style and competitiveness are more common among males (12). The findings are consistent with the findings of Amir and Jelas (2010) which showed that males get higher scores in independent and avoidant scales. In their study, females’ scores in collaborative, dependent, participative and competitive scales were significantly higher than those for males (11). Gujjar and Tabassum (2011) also found similar results but the males’ and females’ scores were not significantly different in avoidant scale (12). Hamidah, Sarina, & Kamaruzaman (2009) also showed that females have higher scores in collaborative, participative, competitive and dependent styles (15).

Mahamod et al. (2010) also found that females use the collaborative, dependent, and participative styles more than males do. In this research, males’ scores in dependent, avoidant and competitive were higher than that of females. The findings of this research also showed that there was no significant difference in Grasha-Riechmann learning styles between native and non-native students (13). Kraft (1976) and O’Faithaigh (2000) showed that males adopt independent and competitive styles more than females do because females naturally experience fear of failure and thus they are dependent on the teachers. Although female students had higher scores in collaborative, participative, dependent and competitive learning styles than males, this difference was not significant (15).

According to the results of the learning styles’ differences in the fields of humanities and science, Fuhrman and Grasha (1983) state that learning styles of participation are influenced by the more underlying characteristics of the personality which may be involved in choosing the field of study. Thus, preferring specific learning styles and tending to choose the specific fields of study may have a common reason. Hence, it is likely that people who have extroversion personality, for example, will choose majors which require interaction with other people. On the other hand, since learning styles are not fixed, they can vary depending on environmental conditions; therefore, in the majors providing more teamwork and collaboration, individuals will have orientation to cooperative and participative styles little by little, and even this may cause them to lose their independence (9).

## Conclusion

The results in relation to learning styles of participation and academic achievement have demonstrated that communication styles will affect learning styles of participation. In Cho et al. (2007), students who had strong friendship networks and communication styles tended to use more cooperative learning and be more successful in using it. As a result, these students gained the best academic outcomes (16). The findings of the present study also showed that science students had higher scores in collaborative, participative, dependent and competitive styles than humanities students. Each of the four styles involves interaction with others (even in the form of comparison in the competitive style). Although it seems there is more interaction in the humanities, actually doing group work and collaborative projects is more common among science students and it may be the reason for the higher scores in these scales for science students.

**Limitation and assumption**

Learning styles can be affected by personality traits, but this variable wasn’t controlled in this study. So, to generalize the study’s results, it should be considered.
